# MRI-based radiomics to predict response in locally advanced rectal cancer: comparison of manual and automatic segmentation on external validation in a multicentre study

**DOI:** 10.1186/s41747-022-00272-2

**Published:** 2022-05-03

**Authors:** Arianna Defeudis, Simone Mazzetti, Jovana Panic, Monica Micilotta, Lorenzo Vassallo, Giuliana Giannetto, Marco Gatti, Riccardo Faletti, Stefano Cirillo, Daniele Regge, Valentina Giannini

**Affiliations:** 1 Department of Radiology, Candiolo Cancer Institute, FPO-IRCCS, Candiolo, Italy; 2 Department of Surgical Sciences, University of Turin, Turin, Italy; 3 Radiology Unit, SS Annunziata Savigliano Hospital, Cuneo, Italy; 4 Politecnico di Torino, Electronic and Telecommunication Department (DET), Turin, Italy; 5 Mauriziano hospital, Turin, Italy; 6 Radiology Unit, Department of Surgical Sciences, University of Turin, Turin, Italy

**Keywords:** Artificial intelligence, Machine learning, Multiparametric magnetic resonance imaging, Neoadjuvant therapy, Rectal neoplasms

## Abstract

**Background:**

Pathological complete response after neoadjuvant chemoradiotherapy in locally advanced rectal cancer (LARC) is achieved in 15–30% of cases. Our aim was to implement and externally validate a magnetic resonance imaging (MRI)-based radiomics pipeline to predict response to treatment and to investigate the impact of manual and automatic segmentations on the radiomics models.

**Methods:**

Ninety-five patients with stage II/III LARC who underwent multiparametric MRI before chemoradiotherapy and surgical treatment were enrolled from three institutions. Patients were classified as responders if tumour regression grade was 1 or 2 and nonresponders otherwise. Sixty-seven patients composed the construction dataset, while 28 the external validation. Tumour volumes were manually and automatically segmented using a U-net algorithm. Three approaches for feature selection were tested and combined with four machine learning classifiers.

**Results:**

Using manual segmentation, the best result reached an accuracy of 68% on the validation set, with sensitivity 60%, specificity 77%, negative predictive value (NPV) 63%, and positive predictive value (PPV) 75%. The automatic segmentation achieved an accuracy of 75% on the validation set, with sensitivity 80%, specificity 69%, and both NPV and PPV 75%. Sensitivity and NPV on the validation set were significantly higher (*p* = 0.047) for the automatic *versus* manual segmentation.

**Conclusion:**

Our study showed that radiomics models can pave the way to help clinicians in the prediction of tumour response to chemoradiotherapy of LARC and to personalise per-patient treatment. The results from the external validation dataset are promising for further research into radiomics approaches using both manual and automatic segmentations.

**Supplementary Information:**

The online version contains supplementary material available at 10.1186/s41747-022-00272-2.

## Key points

• We implemented and validated a promising radiomics model to predict response of locally advanced rectal cancers (LARC) to neoadjuvant chemoradiotherapy in a multicentre dataset.

• To provide a robust model for further clinical applications, we externally validated the model, using patients from a different centre, who underwent magnetic resonance imaging with various scanners and protocols.

• Results on both training and validation datasets were promising and showed that the model could be generalisable.

• Automatic segmentations reached a significantly higher accuracy on the validation set, compared to the manual segmentation, prompting the use of the automatic pipeline on a larger validation cohort.

## Background

Colorectal cancer is the third most common cancer and the second leading cause of cancer death worldwide, representing a tenth of cancer cases and deaths (excluding nonmelanoma skin cancer) [1]. Nearly 40% of the new colorectal tumours are in the rectum, with a proportion of locally advanced rectal cancer (LARC) diagnosis, *i.e.*, T3 or T4 tumours and no nodal involvement or any T and nodal involvement [2, 3]. LARC is increasingly being recognised as a heterogeneous disease, being challenging to cure and with potential different responses to similar treatments [2]. The standard of care is a multimodal approach incorporating neoadjuvant chemoradiotherapy, followed by total mesorectal excision and adjuvant fluoropyrimidine-based chemotherapy. This approach has reduced the rates of local recurrence, but without significant effects on the overall survival [3, 4]. Indeed, only 15–20% of LARC patients achieve a pathological complete response [5] and benefit from alternative treatment strategies rather than radical surgery, maintaining a better quality of life without compromising tumour control [6, 7]. Some patients may not achieve any downstaging of the tumour or even show disease progression [8]; for these nonresponder patients, the side effects of neoadjuvant chemoradiotherapy may outweigh its benefits and different treatment strategies should be considered [9].

Radiomics has recently drawn considerable interest as a potential predictive tool for treatment outcomes, for its important applications in personalised medicine [10]. Once radiomics will be included in the clinical practice, it could help in improving the management of patients with LARC, providing tailored treatments, avoiding unnecessary toxicity to patient predicted unlikely to respond, and consequently anticipating the radical treatment or switching to an intensified treatment when necessary. Recent studies have explored the potential to predict long-term survival of patients with LARC [11], to discriminate different stages of rectal cancer [12], and to predict response to neoadjuvant chemoradiotherapy to identify patients eligible for less invasive treatments [13–17].

One of the critical aspects when dealing with radiomics is tumour segmentation, a process which is performed mostly manually or semiautomatically [18]. This has several drawbacks since it is a time-consuming process and prone to inter-observer variability, which may hamper the reproducibility of radiomics analysis [19, 20]. On the other hand, automatic segmentation techniques based on deep learning are mainstream in the research fields and now are starting to be considered in clinical trials showing improvements in image classification predictions and recognition tasks [21].

This study aims to implement and externally validate a radiomics pipeline to predict neoadjuvant chemoradiotherapy response in patients with LARC, also exploring the impact of manual and automatic segmentations on the radiomics prediction models.

## Methods

### Study design and dataset

We designed and implemented a multicentre retrospective study including patients with stage II/III LARC who underwent either neoadjuvant chemoradiotherapy (capecitabine or 5-FU or CAPEOX or FOLFOX) or radiotherapy only followed by total mesorectal excision from October 2010 to December 2018. Patients were enrolled from three different institutions (centre A: Candiolo Cancer Institute, FPO-IRCCS; centre B: AO Ordine Mauriziano, Torino; centre C: AOU Città della Salute e della Scienza, Torino). Inclusion criteria were (a) biopsy-confirmed stage II/III LARC; (b) multiparametric magnetic resonance imaging (mpMRI) performed before neoadjuvant chemoradiotherapy, including at least an axial T2-weighted (T2w) and axial diffusion-weighted imaging (DWI); and (c) assessment of the tumour regression rate (TRG). One hundred twelve patients satisfied the inclusion criteria. Seventeen were excluded due to either strong mpMRI artifacts (*n* = 15) or misalignment between T2w imaging and DWI (*n* = 2). The final study sample thus included 95 patients.

Patients from both centres A (*n* = 44) and B (*n* = 23) were randomly divided into training (70%) and test (30%) sets, from now on called *construction set*, while the 28 patients from centre C composed the *external validation set*.

### Reference standard

Resected tumours were evaluated by three different experienced pathologists, one for each centre, blinded to clinical information and mpMRI findings. The TRG was assessed according to the Mandard classification [22]. Patients were classified into two different classes: responders if TRG was 1 or 2 and nonresponders if TRG was equal to or greater than 3.

### mpMRI protocol

mpMRI were acquired using different 1.5-T scanners, as following details: centre A: HDx Signa Excite (GE HealthCare, Milwaukee, WI, USA), using an 8-channel phased-array surface coil or Optima MR450w (GE HealthCare, Milwaukee, WI, USA), with a 32-channel phased-array surface coil; centre B: Ingenia (Philips Medical Systems, Eindhoven, The Netherlands), using a 32-channel body phased-array coil; centre C: Achieva (Philips Medical Systems, Eindhoven, The Netherlands), using a 32-channel body phased-array coil. All mpMRI examinations were performed according to the guidelines for pelvic MRI of rectal cancer [23]. Acquisition parameters for the T2w and DWI sequences used in this study are reported in Table 1.

**Table 1:** Multiparametric MRI acquisition

Parameters		Centre A	Centre B	Centre C
T2w	TR/TE	7,660/110 ms	3,231/90 ms	5,085/100 ms
	Acquisition matrix	416 × 224	320 × 311	512 × 512
	Slice thickness	4 mm	3.5 mm	3 mm
	Pixel size	0.43 × 0.43 mm^2^	0.47 × 0.47 mm^2^	0.8 × 0.8 mm^2^
	FOV	220 mm × 220 mm	240 mm × 240 mm	250 mm × 250 mm
	Flip angle	90°	90°	90°
DWI	TR/TE	2,000/87 ms	4,011/91 ms	2,694/68 ms
	Acquisition matrix	96 × 128	100 × 98	124 × 101
	Slice thickness	4 mm	3.5 mm	3 mm
	Pixel size	0.86 × 0.86 mm^2^	1.88 × 1.88 mm^2^	2.8 × 2.8 mm^2^
	FOV	220 mm × 220 mm	240 mm × 240 mm	345 mm × 345 mm
	Flip angle	90°	90°	90°
	*b*-value max	1,200 s/mm^2^	1,000 s/mm^2^	1,000 s/mm^2^

*FOV* Field of view, *ms* Millisecond, *TR/TE* Repetition time/time to echo

### Manual and automatic segmentations

**Fig. 1:**
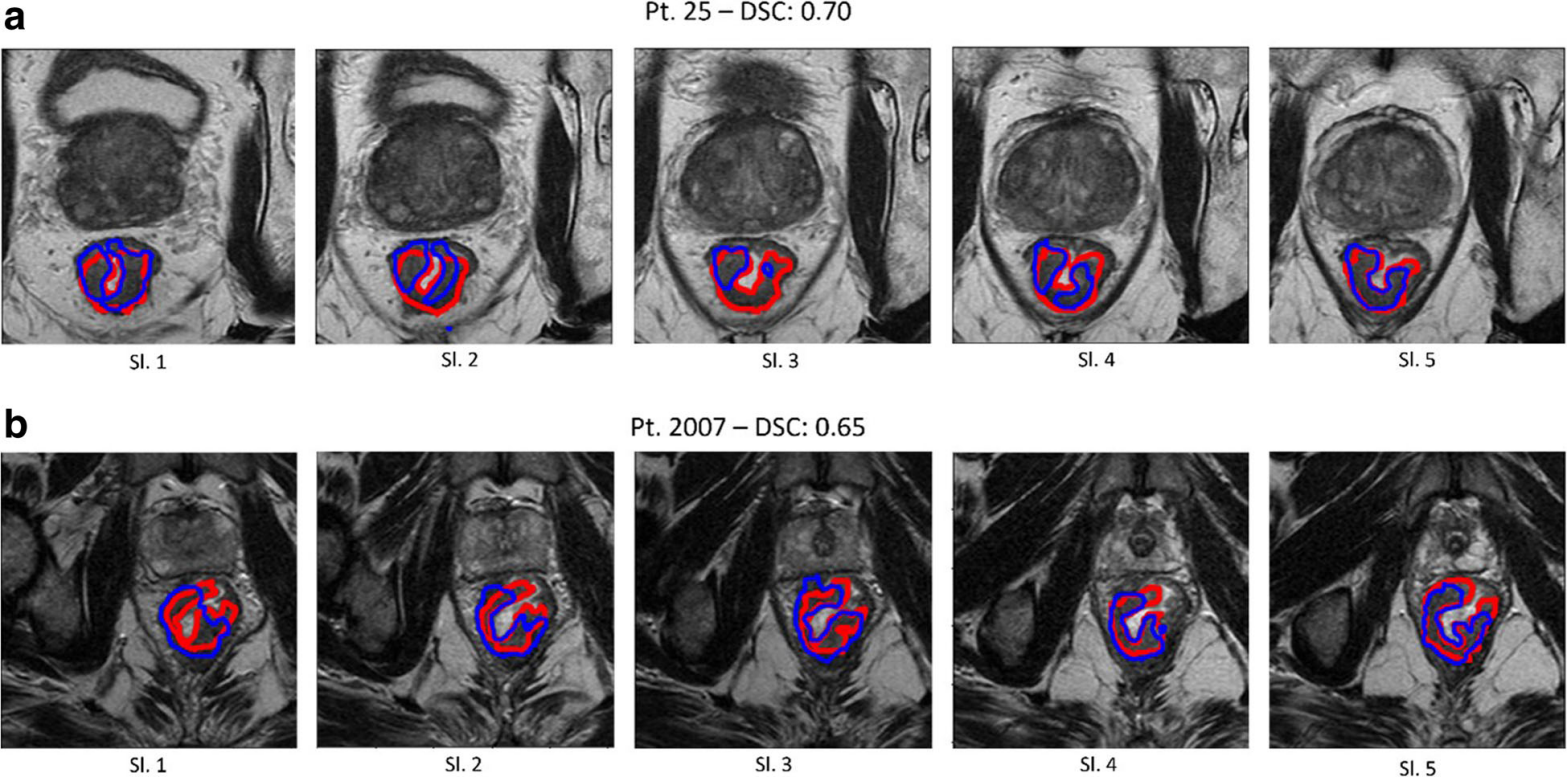
Examples of automatic segmentations of rectal cancer using the fully convolutional network model (blue line) and manual segmentation (red line) in different slices of two patients. First row: patient no. 25 with a DSC of 0.70; second row: patient no. 2007 with a DSC of 0.65. *DSC* Dice similarity coefficient, *Pt* Patient, *Sl.* Slice

Two radiology residents with up to 3 years of experience and one radiologist with 5 years of experience in reporting mpMRI manually segmented rectal tumours on axial T2w imaging using a 3D Slicer (v. 4.10.1, National Institutes of Health, USA). All acquired mpMRI sequences were available to the radiologists during the segmentation process. We chose not to segment the outer edges of the tumours to avoid potential inclusion of desmoplastic striae, areas of extramural vascular invasion, or lymph nodes contiguous to the lesion. Manual segmentations were reviewed by two experienced radiologists with more than 15 years of experience in oncologic MRI. All tumours were also segmented using a previously developed deep learning algorithm, based on fully convolutional networks. The algorithm consists of a pre-processing step to normalise and highlight the tumour area on apparent diffusion coefficient (ADC) maps, followed by segmentation of the tumour on the T2w imaging. Two examples of both manual and automatic three-dimensional segmentations in the same patient are shown in Fig. 1, one from the construction set (patient no. 25. Fig. 1a) and one from the validation set (patient no. 2007, Fig. 1b).

### Feature extraction

Manual and automatic segmentation masks were applied to both T2w and ADC images to extract the corresponding tumour volumes. Texture features were extracted using the *Pyradiomics* package, compliant with the Image Biomarker Standardization Initiative [24], following its flowchart for the extraction steps. We started with the original T2w images, without applying any de-noising Gaussian filter that could introduce recreated and fake information [25], and removed outliers, re-segmenting the masks between the 1st and the 99th percentile of the region of interest (ROI). The voxels outside the range were subsequently excluded from the mask.

Discretisation of image intensities inside the ROI is often required to make the calculation of texture features tractable [25]. We discretised the ROI using a fixed number of bins. This fixed number introduces a normalising effect which may be beneficial when intensity units are arbitrary and when contrast is considered important [25]. We chose 64 bins as a compromise solution: it is a common choice for image quantification in radiomics analysis and it makes possible to explore the whole range of tumour signal intensities.

We extracted 157 features (77 from T2w and 80 from ADC maps):

1. ROI volume (mm^3^) from T2w images
2. 4 intensity-based statistics from ADC maps
3. 50 from GreyLevelCo-occurrence Matrix (GLCM)
4. 32 from GrayLevelRunLength Matrix (GLRLM)
5. 32 from GrayLevelSizeZone Matrix (GLSZM)
6. 10 from NeighborhoodGray-ToneDifference Matrix (NGTDM)
7. 28 from Gray Level Dependence Matrix (GLDM)

The intensity-based statistics were extracted from ADC maps only, since T2w acquisitions may suffer from high variability between scanners and acquisition protocols. For the ADC maps, we considered the first-order features, without any normalisation step, according to the fact that the ADC is already an intrinsic normalised sequence, with a substantial degree of normalisation [26].

Features were calculated for each single slice and then averaged to enable the method to be rotationally invariant (2.5 averaged method for feature extraction, according to the *Pyradiomics* guideline [24]). The distance between two neighbouring voxels was considered equal to one. Details of image processing and features extraction are provided in Supplementary Table 1.

#### Feature selection

Feature selection (FS) is a process of selecting an optimal subset of input variables, excluding irrelevant or redundant data that might cause overfitting and/or be a source of noise for the classifier [27]. In this study, we compared three different FS algorithms: (a) ranking method, (b) affinity propagation, and (c) minimum redundancy maximum relevance. Before applying a FS method, features were normalised using the min-max scaling, choosing the minimum and maximum values on the construction set.

- *Ranking method*. For the training set data, we calculated for each feature the area under the receiver operating characteristics curve (AUC) to estimate its ability to discriminate responders from nonresponders. We then computed the Pearson’s correlation matrix between all features to assess which pairs of features were highly correlated. When a couple of features showed a Pearson’s linear correlation ≥ 0.85, we discarded the features having the lowest AUC. To improve stability and avoid bias, this selection was repeated 100 times, using randomly re-composed training sets (70% of the patients from centres A and B). Features selected at least 70 times were included in the subsequent analysis. Once all non-relevant features were removed, different classifiers were fed with the remaining features ordered by their AUC values. To avoid overfitting, we used a k-fold cross-validation (*k* = 6), consisting in partitioning the dataset into k folds and performing training on all but one-fold, and testing on the left-out fold. This procedure was repeated until each fold was used. The performance of each classifier was then averaged to calculate the mean accuracy of all sub-training sets and the accuracy obtained on the test set. Finally, we selected a threshold on the number of features based on the point of overfitting, defined as the point in which accuracy on the training set keeps increasing while accuracy on the test set starts decreasing.
- *Affinity propagation*. This clustering algorithm does not require the number of clusters to be set a priori. The algorithm associates each feature with an exemplary one: all elements with the same specimen constitute a cluster. In simpler terms, each element sends a message to all the others informing about its affinity towards them. In turn, the other elements respond to the sender informing about their association availability. This exchange of messages continues until each element is associated with a single exemplar (*message passing*). The FS is made by taking only the exemplars as the optimal feature subset [28].
- *Minimum redundancy maximum relevance*. This method searches for a subset composed of features that are minimally related to one another while maximising the prediction of the outcome. The MATLAB function used, called *fsmrmr*, ranks all features in descending order based on their mutual information quotient value. It assigns a score to each feature, as high as the importance of the contribution that the feature gives to the subset of selected ones. The function works iteratively. At each iteration, it identifies the best feature (according to the score) and adds it to the basket of selected features. The next best feature is the one with the highest score. In this way, the optimal subset will be composed of the first *N* features with the highest mutual information quotient.

#### Development and optimisation of classifiers

The previously described FS methods were combined with four different classifiers: support vector machine (SVM), Bayesian model, ensemble learning methods, and logistic regression. Each model was implemented and optimised using the construction set and then validated on an external dataset, using both manual and automatic masks to assess how segmentation affects the results. Only models which reached a minimum accuracy of 0.5 on the construction dataset were used in the next steps.

The SVM performances were optimised by modifying the box constraint (from 1 to 50) and the kernel function (polynomial, linear, Gaussian).

For the ensemble learning, we tested the bagging and the boosting aggregation. Bagging involves having each model in the ensemble vote with equal weight. To promote model variance, bagging trains each model in the ensemble using a randomly drawn subset of the training set. We used the random forest algorithm that combines random decision trees with bagging. For boosting, we used the *Adaboost* MATLAB function, involving incrementally building an ensemble by training each new model instance to emphasise the training instances that previous models misclassified.

To optimise the logistic regression performances, we applied both the stepwise regression method and the generalised linear regression model. The former searches for terms to add to or remove from the model based on the *p*-value of the *F*-statistics. The *p*-Enter value was changed from 0.05 to 0.1 and the *p*-Remove value from 0.2 to 0.3.

Last, for the Bayesian model classifier, we used the MATLAB function *fitcnb*, and no optimisation steps were performed.

### Validation and statistical analysis

Once all models were optimised on the construction set, we selected the two models (one for the manual and the other for automatic segmentation) that reached the highest accuracy and positive predictive value in the prediction of responder patients, unless clear overfitting occurred (AUC ≥ 0.99). These two models were then validated on the validation dataset.

We also performed a hybrid validation, *i.e.*, the best manual model, trained with manual masks, was validated using the automatic masks and, *vice versa*, the best automatic model, trained with automatic masks, was validated using the manual masks. We performed an error analysis between manual and automatic models on the hybrid validation dataset.

We also performed the Mann-Whitney test to evaluate (i) differences between volumes of correct and misclassified tumours for both pipelines and (ii) differences between dice similarity coefficient (DCS) of correct and incorrect classifications for the automatic pipeline. Accuracy, sensitivity, specificity, negative predictive value, and positive predictive value were assessed on both construction and validation datasets. Sensitivity was defined as the number of correctly classified responder patients over the total number of responder cases, and specificity as the number of correctly classified nonresponder patients over the total number of responder cases. Results of the two best models from manual and automatic segmentations were compared using the *t*-test [29].

## Results

### Patient dataset

The final dataset of 95 patients included 58 men and 37 women with an average age of 64 years (range 35−83 years). After total mesorectal excision and pathological evaluation, 42 cases were classified responders (16 with TRG = 1 and 26 with TRG = 2), while the remaining 53 were classified nonresponders (26 with TRG = 3 and 27 with TRG = 4). The construction dataset included 67 patients (27 responders and 40 nonresponders), while the validation set was composed of 28 patients entirely from centre C (15 responders and 13 nonresponders).

In the automatic segmentation analysis, we further excluded 4/67 (6%) patients of the construction set (3 from centre A and 1 from centre B), because tumours were not identified by the segmentation algorithm (*i.e.*, DSC < 0.20).

**Fig. 2:**
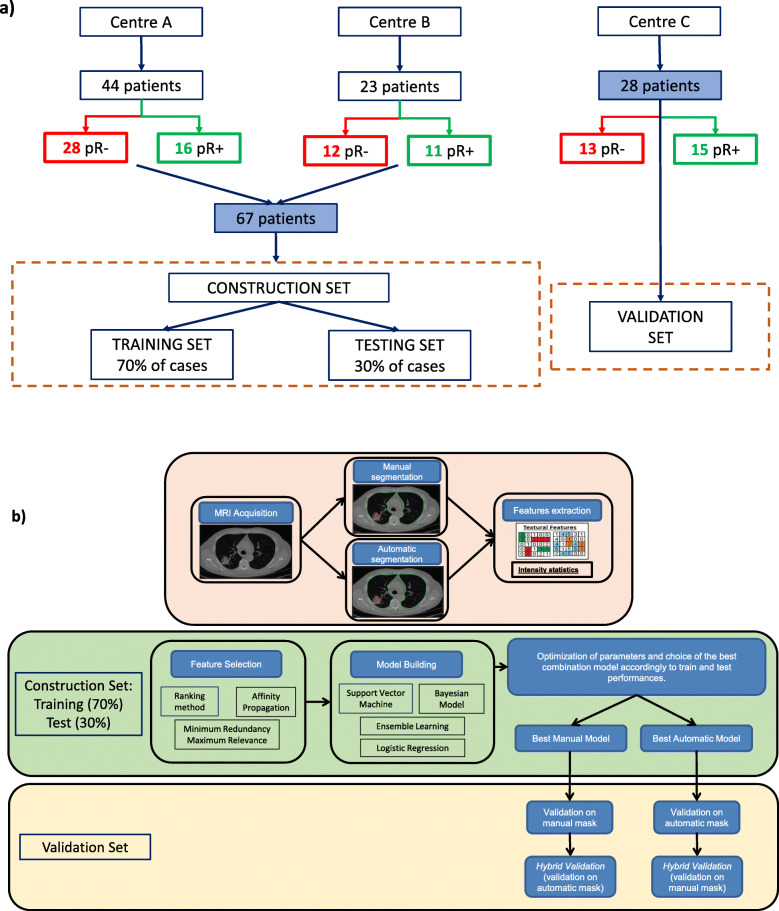
Flowchart explaining the dataset subdivision (**a**). Flowchart illustrating the radiomics pipeline (**b**). *pR* Nonresponders, *pR+* Responders

Patient and tumour data are summarised in Table 2. Figure 2 shows a flowchart detailing the datasets and their subdivision into training, test, and validation sets (Fig. 2a) and the radiomics pipeline (Fig. 2b).

**Table 2:** Patient and tumour data

	Total	Construction	Validation
Number of patients	95	67	28
Number of patients per centre			
Centre A	44	44	–
Centre B	23	23	–
Centre C	28	–	28
Sex	58 M 37 F	43 M 24 F	15 M 13 F
Median age, years [IQR]	64 [34–86]	64 [34–86]	64 [35–83]
TRG			
1	16	9	7
2	26	18	8
3	26	18	8
4	27	22	5
5	0	0	0
Median of tumour volume (cc) [IQR]	21.3 [2.8–232.2]	23.8 [2.8–232.2]	15.4 [3.5–66.7]

*IQR* Interquartile range, *F* Female, *M* Male, *TRG* Tumour regression grade

### Feature selection and classification

#### Manual segmentation

Table 3 shows the results obtained from all combinations of FS and classification methods that reached an accuracy greater than 0.65 on the construction set using manual segmentations. The best SVM model was obtained with polynomial kernel and box constrain of 10, the best ensemble learning results were obtained with the *AdaBoost* aggregation function, while the best results for logistic regression were obtained using a stepwise method with *p*-Enter < 0.1 and *p*-Remove > 0.2.

**Table 3:** Performances for the manual model

	Construction set: centre A + centre B					
	AUC (95% CI)	ACC % (95% CI) [rate]	SE % (95% CI) [rate]	SP % (95% CI) [rate]	NPV % (95% CI) [rate]	PPV % (95% CI) [rate]
MRMR+EL (AdaBoost)	1.00 (93–100)	99 (92–100)	100 (97–100)	98 (87–100)	100 (80–99)	96 (95–100)
		[66/67]	[39/39]	[27/28]	[27/27]	[39/40]
Ranking+EL (Bag)	0.99 (92–100)	94 (85–98)	97 (80–98)	90 (81–100)	96 (74–96)	93 (84–100)
		[63/67]	[37/38]	[26/29]	[26/27]	[37/40]
Ranking+SVM Gaussian	0.87 (79–95)	81 (69–89)	93 (76–99)	73 (67–88)	94 (79–98)	69 (58–79)
		[54/67]	[25/27]	[29/40]	[29/31]	[25/36]
Ranking+LR stepwise	0.69 (67–85)	80 (69–89) [54/67]	89 (71–98) [24/27]	75 (59–87) [30/40]	91 (77–97) [30/33]	70 (57–80) [24/34]
**Ranking+SVM polynomial**	0.90 (82–97)	83 (71–90) [55/67]	85 (66–96) [23/27]	80 (64–91) [32/40]	89 (76–95) [32/36]	74 (60–84) [23/31]
	**Validation set: centre C**					
Ranking+SVM polynomial	0.61 (52–74)	68 (48–84) [19/28]	60 (32–83) [9/15]	77 (47–95) [10/13]	63 (46–76) [10/16]	75 (60–90) [9/12]

*AUC* Area under the curve, *ACC* Accuracy, *NPV* Negative predictive value, *PPV* Positive predictive value, *SE* Sensitivity, *SP* Specificity

We excluded the first two solutions because of overfitting (see Supplementary Table 2). Among the remaining three models, we chose the Ranking approach FS method (which selected 30 features, *i.e.*, ROI volume, 14 GLCM, 9 GLRLM, 6 GLSZM, of whom 4 from the ADC map and 26 from the T2w sequence) with the polynomial SVM classifier. Selected features for the best manual model are reported in Supplementary Table 3, while results of the best model on the validation set are reported in Table 3.

#### Automatic segmentation

The automatic segmentation algorithm showed performances of a median precision of 0.83 (training set), 0.77 (test set), and 0.61 (validation set) in segmenting tumour. In the automatic pipeline, we discarded from the training set all tumours that did not reach a DSC higher than 0.2 compared to the manual segmentation, since these cases were considered false negatives of the segmentation algorithm.

Table 4 shows the results obtained from all combinations of FS and classification methods that reached an accuracy statistically greater than 0.65 using the automatic segmentations. The best SVM model was obtained with a Gaussian kernel function and box constrain of 8, the best ensemble learning results were obtained with the *bagging aggregation* function, and the best results for logistic regression were obtained using a stepwise method with *p*-Enter < 0.1 and *p*-Remove > 0.2.

**Table 4:** Performances for the automatic model

	Construction set: centre A + centre B					
	AUC (95% CI)	ACC % (95% CI) [rate]	SE % (95% CI) [rate]	SP % (95% CI) [rate]	NPV % (95% CI) [rate]	PPV % (95% CI) [rate]
AP+Bayes	0.75 (67–83)	72 (59–82) [45/63]	74 (54–89) [20/27]	69 (52–84) [25/36]	77 (65–87) [25/32]	65 (51–76) [20/31]
MRMR+Bayes	0.78 (69–85)	74 (60–83) [46/63]	81 (62–94) [22/27]	67 (49–81) [24/36]	83 (68–92) [24/29]	65 (44–70) [22/34]
Ranking+EL (Bag)	0.90 (87–95)	68 (55–79)	96 (81–99)	47 (30–65)	94 (71–99)	58 (50–65)
		[43/63]	[26/27]	[17/36]	[17/18]	[26/45]
AP+SVM polynomial	0.70 (64–82)	67 (54–78)	81 (62–94)	56 (38–72)	80 (63–90)	58 (48–67)
		[42/63]	[22/27]	[20/36]	[20/25]	[22/38]
Ranking+SVM linear	0.83 (78–90)	74 (62–85)	70 (50–86)	78 (66–87)	78 (66–87)	70 (55–82)
		[47/63]	[19/27]	[28/36]	[28/36]	[19/27]
**Ranking+SVM Gaussian**	0.86 (78–94)	78 (66–87) [49/63]	81 (62–94) [22/27]	75 (58–88) [27/36]	84 (71–92) [27/32]	71 (57–82) [22/31]
	**Validation set: centre C**					
Ranking+SVM Gaussian	0.81 (60–89)	75 (53–88) [21/28]	80 (50–95) [12/15]	69 (37–90) [9/13]	75 (54–86) [9/12]	75 (55–83) [12/16]

*AUC* Area under the curve, *ACC* Accuracy, *NPV* Negative predictive value, *PPV* Positive predictive value, *SE* Sensitivity, *SP* Specificity

No overfitting was found in the automatic segmentation approach. The best results were obtained with the Ranking method (which selected 27 features: ROI volume, 13 GLCM, 11 GLRLM, 2 GLSZM, of whom 3 from the ADC map and 24 from the T2 sequence) combined with a Gaussian SVM classifier. Selected features are listed in Supplementary Table 4.

### Comparison between manual and automatic segmentation

The best results using manual and automatic segmentations were obtained using the Ranking method and SVM, polynomial for the manual, and Gaussian for the automatic segmentation. Performances reached an accuracy of 68% (manual) and 75% (automatic) in the external validation dataset (*p* = 0.12). The automatic method reached higher performances in sensitivity (80% *versus* 60%, *p* = 0.047) and negative predictive value (75% *versus* 63%, *p* = 0.045), while the manual approach reached higher specificity on the validation set (77% *versus* 69%, *p* = 0.083). The selected features in common between the two best models are 2 from the ADC maps (GreyLevelVariance_glszm and SRHGE_glrlm) and 19 from the T2w sequence, listed in Supplementary Tables 3 and 4.

**Fig. 3:**
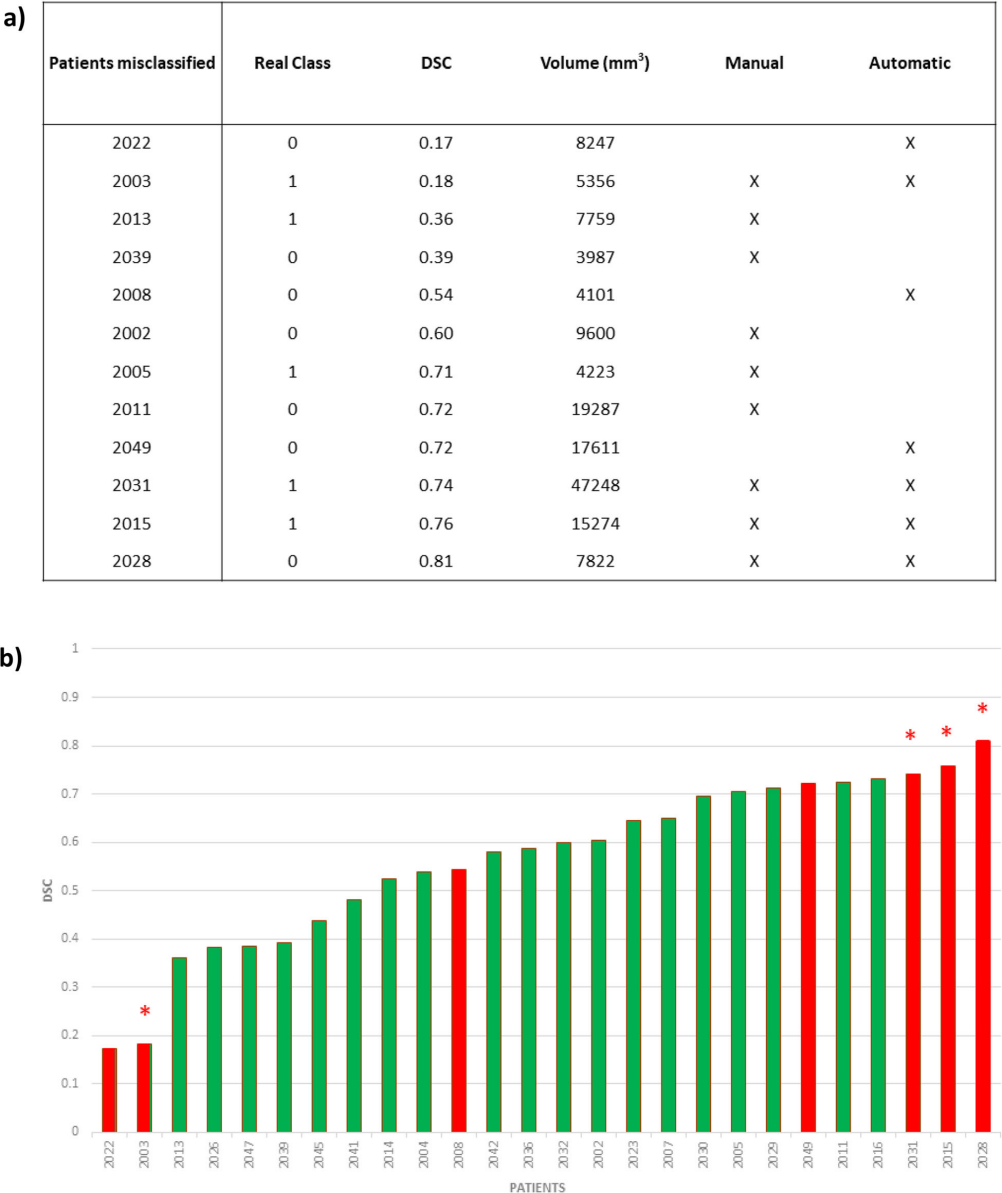
Error analysis. Patients in the validation dataset misclassified by either manual, automatic, or both approaches, sorted by DSC (**a**). **b** Waterfall diagram showing DSC distribution across the validation set for the automatic approach (**b**). Red bars are the automatic model misclassified errors and green bars the correct ones. Red * highlights patients misclassified by both approaches. *DSC* Dice similarity coefficient

We performed an error analysis between manual and automatic models for the validation dataset. Nine of 28 patients were misclassified by the manual segmentation model, seven patients were misclassified by the automatic segmentation model, and four patients were misclassified by both models. Figure 3a reports DSC values and tumour volumes. No differences in volume were found between misclassified tumours of the automatic and manual segmentations (*p* = 0.813). In the manual pipeline, there was no significant difference between volumes of correct and misclassified tumours. Similarly, in the automatic pipeline, volumes and DSC values were not statistically different between correctly and misclassified tumours.

**Fig. 4:**
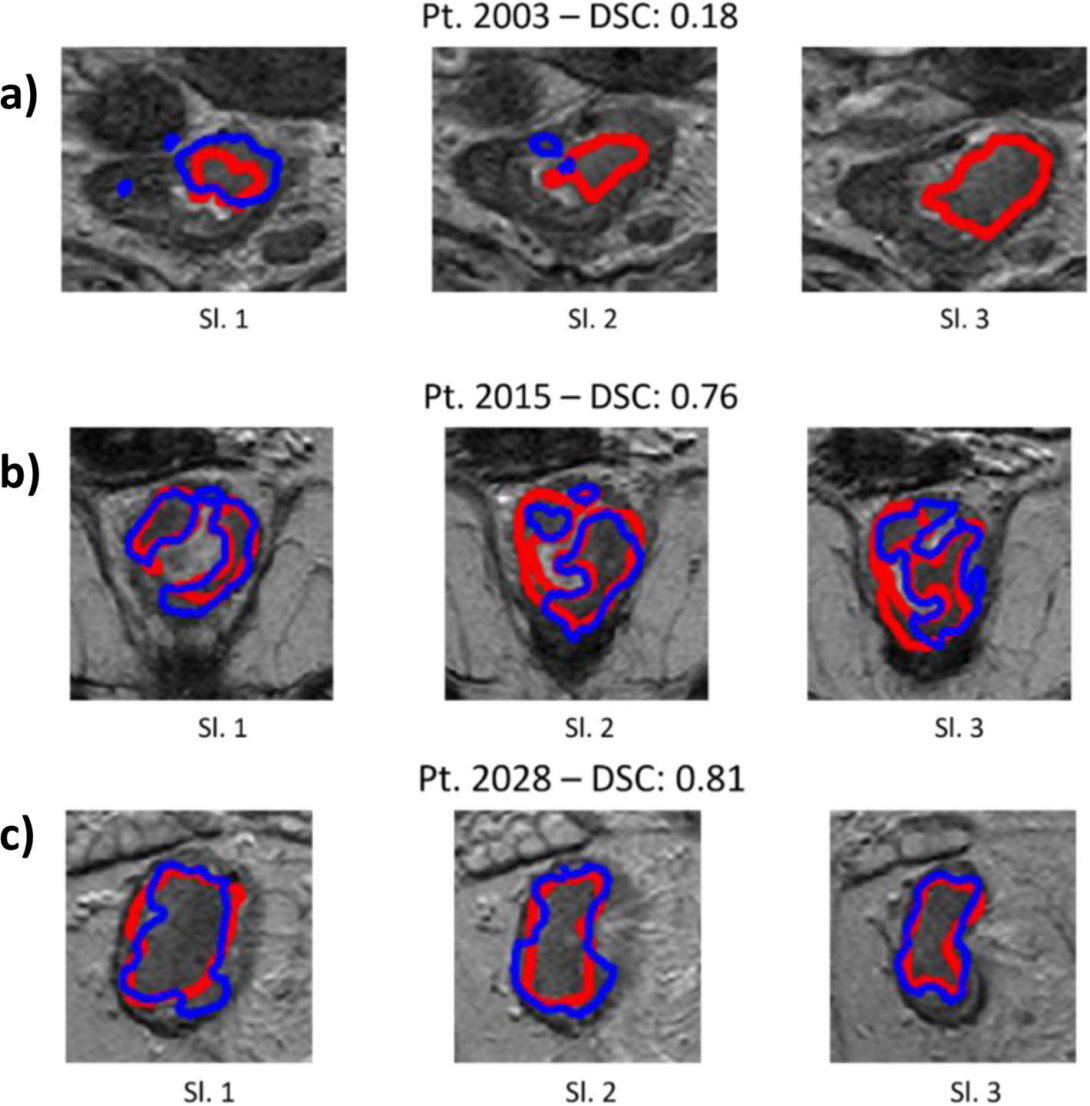
Examples of three patients segmented by internally developed fully convolutional network model (blue line) and the radiologists (red line). Pt. 2003 with DSC of 0.18 (**a**) and Pt. 2015 with DSC of 0.76 (**b**) were wrongly classified by both manual and automatic systems, while Pt. 2028 with DSC of 0.81 (**c**) was wrongly classified only by the automatic model. *DSC* Dice similarity coefficient, *Pt* Patient, *Sl.* Slice

Figure 3b shows the DSC distribution for both manual and automatic segmentations. All tumours with DSC values < 0.3 were misclassified, probably because the segmented area did not include relevant information. However, one case was also misclassified by the manual pipeline, meaning that this patient could have different characteristics in pixel intensity (Fig. 4a). Similarly, among the 4 misclassified tumours with DSC > 0.70, 3 were misclassified also by the manual pipeline (Fig. 4b). Among them, patient no. 2028 was misclassified despite the good segmentation (DSC > 0.8, Fig. 4c), probably due to the intrinsic characteristics of the tumour.

Supplementary Fig. S1 reports the two bar diagrams showing how tumour volume for both manual and automatic segmentations did not influence the prediction in the validation cohort. Errors are equally distributed in all ranges of volume values and there is no statistical correlation between tumour volume and correct classification (*p* = 0.125).

### Hybrid validation

Hybrid results are reported in Table 5. The model *Train_MAN + Val_AUTO* reached higher accuracy values than the results of the manual validation (accuracy on manual validation was 68%, while positive predictive value remained 75%). The model *Train_AUTO + Val_MAN* had sensitivity and negative predictive values too low to be considered. The *Train_MAN + Val_AUTO* model misclassified 7 patients out of 28, while the *Train_AUTO + Val_MAN model* misclassified 10. The performances between the *Train_MAN + Val_AUTO* and the fully automatic approach reached the same performances on the validation set (accuracy 75%). Both made seven misclassification errors, four of them involving the same patients (patient no. 2031, 2015, 2008, and 2049).

**Table 5:** Results on the hybrid validation for the two best models

	Hybrid validation: centre C					
	AUC (95% CI)	ACC % (95% CI) [rate]	SE % (95% CI) [rate]	SP % (95% CI) [rate]	NPV % (95% CI) [rate]	PPV % (95% CI) [rate]
Train_MAN + Val_AUTO	0.69 (60–89)	75 (53–88) [21/28]	80 (50–95) [12/15]	69 (37–90) [9/13]	75 (54–86) [9/12]	75 (55–83) [12/16]
Train_AUTO + Val_MAN	0.62 (52–73)	64 (44–81) [18/28]	47 (21–73) [7/15]	85 (55–98) [11/13]	58 (45–70) [11/19]	78 (47–93) [7/9]

*AUC* Area under the curve, *ACC* Accuracy, *NPV* Negative predictive value, *PPV* Positive predictive value, *SE* Sensitivity, *SP* Specificity

## Discussion

In this study, we developed and compared performances of different machine learning algorithms based on pretreatment mpMRI to predict treatment response in LARC patients. Best performances were obtained using the Ranking approach as feature selection and SVM as classifier for both manual and automatic segmentations. The two best models reached an accuracy of 83% and 78% on the construction dataset and 68% and 75% on the validation dataset, on the manual and automatic pipeline, respectively. Also, the two best models showed a good balance between sensitivity and specificity in the validation set: 60% and 77%, respectively, for the manual model; 80% and 69%, respectively, for the automatic approach.

Results are encouraging and we found similar accuracy on both the external validation datasets, independently of the segmentation method, when the algorithms were implemented using the manual segmentation masks. This finding brings a twofold improvement in knowledge: first, proper manual segmentation is needed to develop a pipeline that would be less sensitive to slightly different segmentations; second, automatic segmentation is an equally effective alternative to the manual approach during the validation phase. This finding could be explained by the fact that radiomics features extracted from “slightly different” parts of the tumour are consistent. Based on this hypothesis, we can also presume that either the manual or automatic masks accurately represent tumour heterogeneity. This data is of key importance when performing large cohort multicentre studies since automatic segmentation is quite unavoidable to bridge the gap between research and clinical applications. Moreover, our analysis demonstrated that misclassified cases are not statistically correlated to tumour volume; however, we found that the variable *ROI volume* was selected as a significant variable by the FS methods in both approaches. Hence, an automatic segmentation algorithm estimating lesion volumes should again be unavoidable when a large group of patients are enrolled.

To the best of our knowledge, this was the first study comparing manual and automatic segmentations in a radiomics study to predict tumour response to neoadjuvant chemoradiotherapy in patients with LARC.

Previously, radiomics was used by Nie et al. [30] and by Horvat et al. [31] to predict treatment response in LARC patients. Nie et al. [30] built a radiomics model using the three mpMRI sequences: T2w, DWI, and dynamic contrast-enhanced, reporting an AUC of 0.89 on a quite small training set (*n* = 48). Horvat et al. [31] focused their work on evaluating the ability of T2w and DWI features to differentiate complete to clinical partial response; the best result was reached using a random forest model that achieved an AUC of 0.93, with a sensitivity of 100%, specificity of 91%, positive predictive value of 72%, and negative predictive value of 100%. However, these two studies lacked a validation cohort, not providing an overfitting analysis of the training performances and so limiting their applicability in clinical practice.

Petkovska et al. [32] used as a validation dataset a different manual segmentation performed by a second radiologist on a subgroup of patients of the training set, reaching an accuracy of 74% and AUC of 0.75. Liu et al. [33] and Shaish et al. [11] performed cross-validation, reaching an AUC of 0.98 and 0.80, respectively. Also, Delli Pizzi et al. [34] reached an AUC of 0.79, performing cross-validation including both features extracted from the core and the border of the tumour. Li et al. [35] and Cui et al. [13] enrolled patients from a single-centre study and performed an internal validation, randomly dividing the dataset into two subgroups, reaching an AUC of 0.87 and 0.95 on the validation cohort, respectively. Only Bulens et al. [36] and Petresc et al. [14] validated their models on an external validation dataset, reaching accuracies of 0.82 and 0.65, respectively, but with a great unbalance between sensitivity and specificity, *i.e.*, sensitivity of 33% and specificity of 97%, and sensitivity of 75% and specificity 60%, respectively.

Our study also presents some limitations. First, it is a retrospective study and selection bias might occur; however, enrolling subsequent patients and having an external validation set could mitigate this bias. The implementation of a prospective clinical trial would be of key importance to further validate our results. Second, the sample size is modest (*n* = 95), especially for the validation set (*n* = 28) and the number of patients excluded, due to mpMRI artifacts, was relatively high (*n* = 15). This might affect the study power and its clinical applicability; however, this preliminary analysis considered patients in a 10-year timespan, whose acquisitions were not optimised for the purpose of radiomics modelling. Nevertheless, results are encouraging, and we are planning to increase the sample size, considering more recent acquisitions with improved MRI protocols and better image quality. Increasing the dataset, along with the refinement of the automatic segmentation algorithm (enhancing DSC values), could lead to a more robust machine learning system. Third, the number of features included in the final models could be rather high, especially when compared to the sample size, leading to the risk of overfitting. However, we believe that using a validation cohort minimised this risk, which would be further reduced increasing the dataset and reanalysing the data in a future study. Last, we classified patients into two different groups, according to their TRG (1, 2 *versus* 3, 4, 5), including the partial response (TRG 3) into the nonresponder class. It could be interesting to consider partial response to treatment as a different class, since the neoadjuvant chemoradiotherapy could provide some benefits to these patients.

As a future step, we will also focus our attention on the integration between radiomics studies and other *omics*, like genomics and pathomics [37, 38]. This is a research topic that should be considered in next-generation multiomics predictive tools.

In conclusion, our study shows that radiomics models can pave the way to help clinicians in the prediction of tumour response to chemoradiotherapy and to personalise per-patient treatment. The results from the external validation dataset are promising for further research into radiomics approaches on both manual and automatic segmentation. Overall, this could represent an innovative starting point for the introduction of an AI tool in helping the decision-making process, to noninvasively select patients eligible, *i.e.*, for organ-preserving strategies or therapies changings. Nevertheless, further studies are needed, including larger datasets from multiple centres, also improving the automatic segmentation process, to fully integrate these methods in a real-world setting.

## Supplementary Information

**Additional file 1.** Supplementary tables and figure

## Data Availability

The datasets generated and/or analysed during the current study are not publicly available because of the terms of the research participant consent but are available from the corresponding author on reasonable request.

## Abbreviations

ADC: Apparent diffusion coefficient
AUC: Area under the curve
DSC: Dice similarity coefficient
DWI: Diffusion-weighted imaging
FS: Feature selection
GLCM: GreyLevelCo-occurrence Matrix
GLRLM: GrayLevelRunLength Matrix
GLSZM: GrayLevelSizeZone Matrix
LARC: Locally advance rectal cancer
mpMRI: Multiparametric magnetic resonance imaging
MRI: Magnetic resonance imaging
ROI: Region of interest
SVM: Support vector machine
T2w: T2-weighted
TRG: Tumour regression grade
