# Supplementary material for: MRI-based radiomics to predict response in locally advanced rectal cancer: comparison of manual and automatic segmentation on external validation in a multicentre study
Source: Eur Radiol Exp. 2022 May 3;6:19. doi: 10.1186/s41747-022-00272-2 (PMC9061921; doi:10.1186/s41747-022-00272-2)
Supplement: Supplementary file 1 — Additional file 1. Supplementary tables and figure [file 41747_2022_272_MOESM1_ESM.docx]

**ELECTRONIC SUPPLEMENTARY MATERIAL**

Supplementary table 1: Reporting Guidelines: from Image processing to features calculation steps.

| **Area** | **Topic** | **Description** |
| --- | --- | --- |
| **Patient** | Region of interest | pelvic zone for rectal cancer staging |
|  | Contrast agent (only for lesion detection) | a 20-mg injection of scopolamine butyl-bromide |
| **Acquisition** | Acquisition protocol | Different acquisition protocols |
|  | Scanner type | HDx Signa Excite, GE HealthCare, Milwaukee, WI, USA, at Candiolo;  Ingenia, Philips Medical Systems, Eindhoven, The Netherlands, at Mauriziano.  Achieva, version 2.6, Philips Medical Systems, Eindhoven, The Netherlands, at Molinette. |
|  | Imaging modality | Magnetic Resonance |
|  | Scan duration | Approximately 40 minutes |
| **Image registration** | Registration method | Rigid registration to align DCE and T2w images and elastic registration to align DWI and T2w images (described in doi:10.1166/jmihi.2015.1518). |
| **Data conversion** | ADC computation | ADC= ln(S_0_/S_1_)/(b_1_-b_0_), where S_0_ and S_1_ are the signal intensity obtained with the b_0_ and b_1_ b values, respectively. |
|  | Other data conversions | - |
| **Post-acquisition processing** | Anti-aliasing | - |
|  | Non-uniformity correction | - |
|  | Intensity normalization | - |
| **Segmentation** | Method | Manual segmentation on T2w |
|  | Conversion to mask | NIFTI |
| **Image Interpolation** | Interpolation method | Trilinear interpolation performed aligning grid centers (rounded to the nearest integer). |
|  | Voxel dimension | 0.5 mm |
| **Image Interpolation** | Interpolation method | Nearest integer interpolation performed aligning grid centers (rounded to the nearest integer). |
|  | Partially masked voxel | Threshold of 0.5 |
| **Re-segmentation** | Method | Between 1^st^ and 99^th^ percentile |
| **Discretization** | Method | Fixed bin number (64 bin) |
| **Image transformation** | Image filter | NONE |
| **Image biomarker computation** | Biomarker set | Intensity-based statistics (from ADC maps), intensity histogram (from ADC maps), GLCM, GLRLM, GLSZM, NGTDM, GLDM (from ADC maps and T2w images) |
|  | IBSI compliance | Yes |
|  | Software availability | Pyradiomics |
| **Image biomarker computation - texture parameters** | Texture matrix aggregation | 2.5 averaged |
|  | Distance weighting | No weighting |
|  | CM symmetry | Symmetric co-occurrence matrices |
|  | CM distance | 1 |
| **List of features** | (T2w only) | ROI volume |
|  | Intensity-based statistics (ADC only) | skewness, kurtosis, intensity kurtosis and intensity variance |
|  | GLCM (for both T2w and ADC) | Joint Max,  Joint Average,  Joint Variance,  Joint Entropy,  Difference Average,  Difference Variance,  Difference Entropy,  Sum Average,  Sum Variance,  Sum Entropy,  Angular Second Moment,  Contrast,  Dissimilarity,  Inverse Difference,  Normalised Inverse Difference,  Inverse Difference Moment,  Normalised Inverse Difference Moment,  Inverse Variance,  Correlation,  Autocorrelation,  Cluster tendency,  Cluster shade  Cluster prominence,  Information Correlation 1,  Information Correlation 2. |
|  | GLRLM (for both T2w and ADC) | Short runs emphasis,  Long runs emphasis,  Low grey level run emphasis,  High grey level run emphasis,  Short run low grey level emphasis,  Short run high grey level emphasis,  Long run low grey level emphasis,  Long run high grey level emphasis,  Grey level non-uniformity,  Normalised grey level non-uniformity,  Run length non-uniformity,  Normalised run length non-uniformity,  Run Percentage,  Grey level Variance,  Run length Variance,  Run Entropy. |
|  | GLSZM (for both T2w and ADC) | Gray Level Non-Uniformity  Gray Level Non-Uniformity Normalized  Gray Level Variance  High Gray Level Zone Emphasis  Large Area Emphasis  Large Area High Gray Level Emphasis  Large Area Low Gray Level Emphasis  Low Gray Level Zone Emphasis  Size Zone Non-Uniformity  Size Zone Non-Uniformity Normalized  Small Area Emphasis  Small Area High Gray Level Emphasis  Small Area Low Gray Level Emphasis  Zone Entropy |
|  | NGTDM (for both T2w and ADC) | Busyness  Coarseness  Complexity  Contrast  Strength |
|  | GLDM (for both T2w and ADC) | Dependence Entropy  Dependence Non-Uniformity  Dependence Non-Uniformity Normalized  Dependence Variance  Gray Level Non-Uniformity  Gray Level Variance  High Gray Level Emphasis  Large Dependence Emphasis  Large Dependence High Gray Level Emphasis  Large Dependence Low Gray Level Emphasis  Low Gray Level Emphasis  Small Dependence Emphasis  Small Dependence High Gray Level Emphasis  Small Dependence Low Gray Level Emphasis |
|  |  |  |

Supplementary table 2: Results for the manual approach where overfitting occurs.

|  | **Validation set Center C** | | | | | |
| --- | --- | --- | --- | --- | --- | --- |
|  | AUC  (95% CI) | ACC %  (95% CI)  [rate] | SE %  (95% CI)  [rate] | SP %  (95% CI)  [rate] | NPV %  (95% CI)  [rate] | PPV %  (95% CI)  [rate] |
| MRMR+EL (AdaBoost) | 0.60  (39-87) | 66  (48-84) | 87  (60-98) | 46  (19-75) | 75  (52-76) | 65  (52-76) |
|  |  | [19/28] | [13/15] | [6/13] | [6/8] | [13/20] |
| Ranking+EL (Bag) | 0.58  (41-81) | 64  (44-81) | 80  (51-96) | 46  (27-66) | 67  (38-87) | 63  (49-75) |
|  |  | [18/28] | [12/15] | [6/13] | [6/9] | [12/19] |

AUC: area under the curve, ACC: accuracy, NPV: negative predictive value, PPV: positive predictive value, SE: sensitivity, SP: specificity.

Supplementary table 3: List of features selected for the best manual model. All features extracted from the T2 image, expect the ones named _ADC.

| \| roi volume \| \| --- \| \| JointMax_GLCM \| \| JointAverage_GLCM \| \| JointEntropy_GLCM \| \| diffAverage_GLCM \| \| diffVariance_GLCM \| \| diffEntropy_GLCM \| \| sumAverage_GLCM \| \| sumVariance_GLCM_ADC \| \| sumEntropy_GLCM \| \| contrast_GLCM \| \| InverseDifference_GLCM \| \| Autocorrelation_GLCM \| \| clustertendency_GLCM_ADC \| \| infCorr2_GLCM \| \| HGRE_GLRLM \| \| SRLGE_GLRLM \| \| SRHGE_GLRLM_ADC \| \| LRLGE_GLRLM \| \| GLNU_GLRLM \| \| GLNU_norm_GLRLM \| \| RLNU_GLRLM \| \| RLNU_norm_GLRLM \| \| RunEntropy_GLRLM \| \| GrayLevelNonUniformity_glszm \| \| GrayLevelNonUniformityNormalized_glszm \| \| GrayLevelVariance_glszm_ADC \| \| LargeAreaEmphasis_glszm \| \| LargeAreaHighGrayLevelEmphasis_glszm \| \| LargeAreaLowGrayLevelEmphasis_glszm \| |
| --- | --- | --- | --- | --- | --- | --- | --- | --- | --- | --- | --- | --- | --- | --- | --- | --- | --- | --- | --- | --- | --- | --- | --- | --- | --- | --- | --- | --- | --- | --- |

Supplementary table 4: List of features selected for the best automatic model. All features extracted from the T2 image, expect the ones named _ADC.

| roi volume |
| --- |
| JointMax_GLCM |
| JointAverage_GLCM |
| JointEntropy_GLCM |
| diffAverage_GLCM |
| diffVariance_GLCM |
| diffEntropy_GLCM |
| sumAverage_GLCM |
| sumVariance_GLCM |
| sumEntropy_GLCM |
| contrast_GLCM |
| InverseDifference_GLCM_ADC |
| Autocorrelation_GLCM |
| clustertendency_GLCM |
| HGRE_GLRLM |
| SRLGE_GLRLM |
| SRHGE_GLRLM_ADC |
| LRLGE_GLRLM |
| LRHGE_GLRLM |
| GLNU_GLRLM |
| GLNU_norm_GLRLM |
| RLNU_GLRLM_ADC |
| RLNU_norm_GLRLM |
| GreylevelVariance_GLRLM |
| RunEntropy_GLRLM |
| GrayLevelNonUniformity_glszm |
| GrayLevelVariance_glszm_ADC |


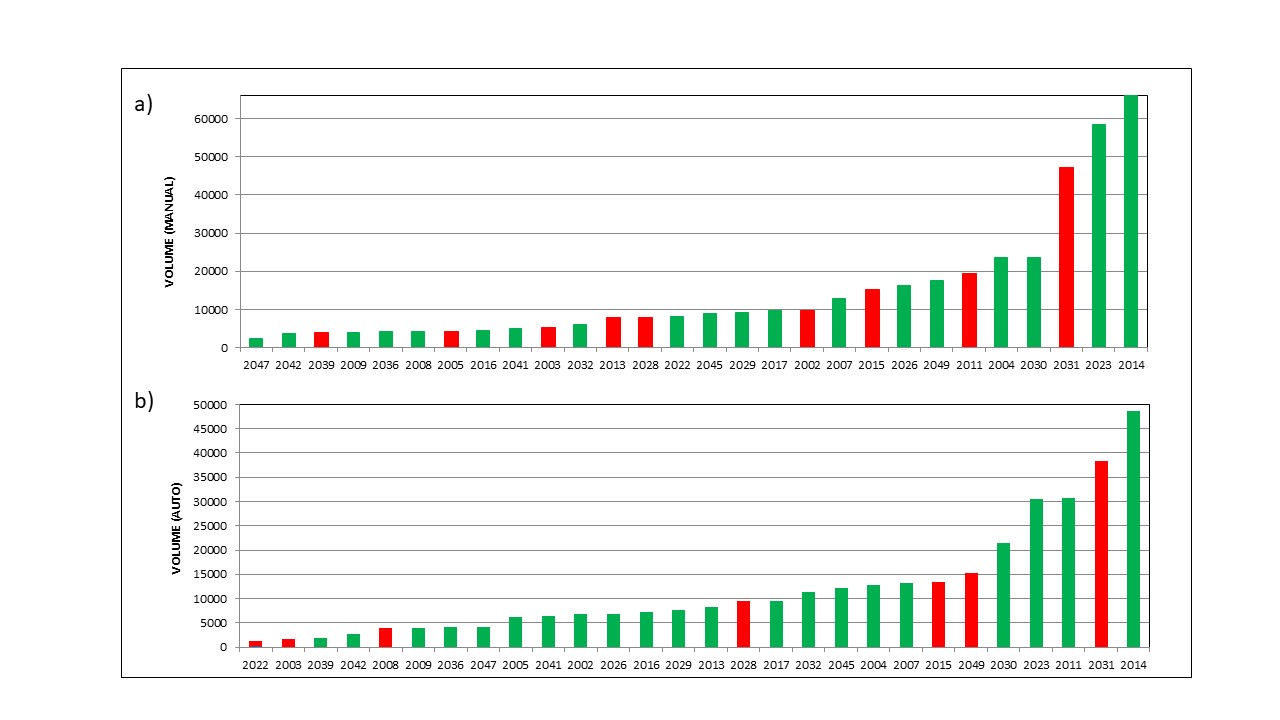
Supplementary Figure 1. Waterfall graph: a) Listed of patients sorted by increasing volume (mm^3^). Red bars are the manual model misclassified errors and green bars the correct ones. b) Listed of patients sorted by increasing volume (mm3). Red bars are the automatic model misclassified errors and green bars the correct ones.
